# Surgical Management of Inherited Breast Cancer: Role of Breast-Conserving Surgery

**DOI:** 10.3390/cancers14133245

**Published:** 2022-07-01

**Authors:** Francesca Magnoni, Virgilio Sacchini, Paolo Veronesi, Beatrice Bianchi, Elisa Bottazzoli, Valentina Tagliaferri, Erica Mazzotta, Giulia Castelnovo, Giulia Deguidi, Elisabetta Maria Cristina Rossi, Giovanni Corso

**Affiliations:** 1 Division of Breast Surgery, IEO European Institute of Oncology, IRCCS, 20041 Milan, Italy; paolo.veronesi@ieo.it (P.V.); beatrice.bianchi@ieo.it (B.B.); elisaileana.bottazzoli@ieo.it (E.B.); valentina.tagliaferri@ieo.it (V.T.); erica.mazzotta@ieo.it (E.M.); giuliacastelnovo186@gmail.com (G.C.); giulia.deguidi@ieo.it (G.D.); elisabettamariacristina.rossi@ieo.it (E.M.C.R.); giovanni.corso@ieo.it (G.C.); 2 Breast Service, Department of Surgery, Memorial Sloan Kettering Cancer Center, New York, NY 10065, USA; sacchinv@mskcc.org; 3 Department of Oncology and Hemato-Oncology, University of Milan, 20122 Milan, Italy

**Keywords:** hereditary breast cancer, *BRCA* gene, *BRCA* mutation, risk-reducing surgery, breast-conserving surgery, nipple-sparing mastectomy, local recurrence, survival, outcome

## Abstract

**Simple Summary:**

Which should be the optimal surgical approach to breast cancer in the presence of high penetrance gene mutation represents a current clinical and scientific issue, lively debated and studied. Does inherited breast cancer always mean bilateral mastectomy? Scientific research is questioning the oncological safety of a conservative surgical approach in hereditary breast cancer. The present narrative review aims to explore the scientific panorama on this faceted and significant theme.

**Abstract:**

Recent studies have demonstrated that hereditary breast cancer (BC) has a prevalence of 5–10% among all BC diagnoses. Nowadays, significant technological advances in the identification of an increasingly broad spectrum of genetic mutations allow for the discovery of an ever-growing number of inherited pathogenic (P) or likely pathogenic (LP) variants of breast cancer susceptibility genes. As the management of BC patients carrying mutations in the *BRCA1/2* genes or other high-penetrance genes is currently a challenge, extensive research is being carried out and a lively scientific debate has been taking place on what the most appropriate local therapy, especially surgical treatment, of patients with inherited BC should be. In many studies, BC outcomes in *BRCA* carriers and non-carriers have been compared. A number of them showed that, when compared with mastectomy, breast-conserving surgery in *BRCA* patients is oncologically safe in terms of overall survival, although an increased risk of ipsilateral recurrence was reported. In these patients, devising a specific therapeutic strategy is an inevitably complex process, as it must take into consideration a series of factors, require a multimodal approach, guarantee personalization, strictly adhere to scientific international guidelines, and consider all available evidence. The present narrative review purposes to identify and illustrate evidence from significant selected studies that discussed those issues, as well as to suggest useful tools to clinicians managing this specific clinical condition in daily clinical practice.

## 1. Introduction

The landscape of breast cancer (BC) associated with hereditary predisposition is currently in considerable evolution thanks to the significant refinements of emerging new technologies in testing genetic susceptibility to cancer [1]. Indeed, scientific research is extensively studying what the better curative strategy should be. Could breast-conserving therapy, defined as breast-conserving surgery combined with radiotherapy, be considered a safe local treatment in *BRCA* mutation carriers? The present narrative review aims to extensively analyze the literature of the last 20 years, the livelier on such issues, as selecting and differentiating scientific sources by type, retrospective studies, and meta-analyses. Thus, it reports the relevant findings of scientific literature on the outcome of the conservative surgical approach to hereditary breast cancer linked to pathogenic mutations in high penetrance genes, focusing on *BRCA1* and *2* genes, potentially helpful in daily clinical practice. Starting from an overview of the current state of the art of inherited mutations linked to breast cancer, it develops through a specialized and technical study of preventive mastectomy, up to the analysis of the novel role of breast-conserving therapy and its oncological safety in *BRCA* carriers.

## 2. Background: Landscape of Inherited Mutations and Breast Cancer

The American Society of Clinical Oncology (ASCO) has long stated that “the recognition and management of individuals with an inherited susceptibility to cancer are core elements of oncology care” [1]. The notion of tumor inheritance, understood as the transmission of the disease from one generation to the next, was first described by Paul Broca in 1866 [2]. The original aim of risk assessment was mainly to offer information regarding second cancer risk and risk to family members [1]. Nowadays, cancer treatment itself often includes knowledge of whether a germline mutation is present [1]. The application to somatic mutation profiling of next-generation sequencing (NGS), which allows a quicker and less expensive characterization of large parts of DNA compared to previous techniques [3,4], has made testing more than one gene at a time a reality [5,6,7,8], with the possibility of incidentally identifying inherited risks [1]. NGS offers the advantage of facilitating the identification of inherited susceptibility to cancer and other diseases either during somatic mutation profiling or through direct germline multigene multiplex panel testing [1]. NGS, therefore, represents a fine additional diagnostic tool in the oncology context. By identifying changes in DNA sequences, it can point out targets for specific therapy and improve the prognosis of the disease [1]. Most patients present with a diagnosis of BC without a known family history of it. Approximately 5–10% of BCs are considered hereditary because of an inherited P or LP variant of a BC susceptibility gene [9]. The discovery of two major BC susceptibility genes, *BRCA1* and *BRCA2*, in 1994 and 1995, respectively, revealed the association between family history and the presence of inherited genetic events that predispose individuals to BC development [10,11]. *BRCA1* and *BRCA2* are two critical tumor suppression genes involved in the repair of double-stranded DNA breaks by homologous recombination [12]. Homologous recombination deficiency (HRD) is the main cause of the increased risk of BC development in *BRCA* mutation carriers.

P or LP variants of *BRCA1* and *BRCA2* include more than 80% of hereditary BC and confer an approximately 50–60% absolute risk of developing it by the age of 80 [13,14]. *BRCA1*/2 mutated BC accounts for 3–12% of all BCs in women, 10–20% of which are triple-negative [15,16]. A genome-wide association study (GWAS) investigating BC risk in *BRCA,* and non-*BRCA* mutation carriers reported it to be higher in some of the populations under study, such as people of Ashkenazi Jewish descent, in *BRCA1* rather than in *BRCA2* mutation carriers, in younger women, in the presence of the 5′ to c.2281 and c.4072 to 3′ *BRCA1* mutations, and also in patients with a positive family history [13]. The pathological features conferred to BC cells by P or LP variants of *BRCA1* and *BRCA2* differ from those found in sporadic BC [17]. *BRCA1* mutations are associated with estrogen-receptor-negative, progesterone-receptor-negative, and HER2-negative (triple-negative) BC with a basal-like gene expression profile [18]. *BRCA2*-associated breast tumors are usually high-grade, estrogen-receptor-positive, and HER2-negative [19,20]. The associated clinical characteristics, moreover, differ, including early-onset diseases, bilateral BC, other synchronous malignancies, especially ovarian cancer, and an increased rate of P53 mutation [17,21]. Thus, the clinical treatment strategy for BC with *BRCA* mutations should be different and personalized in terms of both local and systemic control. In the last two decades, since the *BRCA* genes discovery, significant advances have been made in identifying further germline pathogenic variants of cancer-predisposition gene associated with an increased risk of BC [22,23,24,25].

Recent population-based studies have clarified the risk of BC conferred by many deleterious gene mutations and classified them into high–(causing a threefold or higher increase in the risk of BC relative to the general population), intermediate–(twofold to threefold increase), or low-penetrance (onefold to twofold increase) [24,26].

A recent population-based matched analysis by Hu et al. reported that among women with BC, the prevalence of 12 established BC-predisposition genes (*ATM*, *BARD1*, *BRCA1*, *BRCA2*, *CDH1*, *CHEK2*, *NF1*, *PALB2*, *PTEN*, *RAD51C*, *RAD51C,* and *TP53*) is close to 5% [26]. Indeed, P or LP variants of other BC susceptibility genes, including high-penetrance genes such as *CDH1*, *PALB2*, *PTEN*, *STK11*, and *TP53*, and moderate-penetrance genes such as *ATM*, *CHEK2*, *NBN*, and *NF1*, also confer an increased BC risk [9,22,23,25,27]. These cancer-predisposing variants of not only high- but also moderate-penetrance genes can be largely detected thanks to improvements in sequencing technology and multigene panel testing [28]. Consequently, further genetic variants associated with an increased risk not only of BC but also of ovarian, fallopian tube, colon, melanoma, prostate, and pancreatic cancer were discovered [28]. In general, high-penetrance variants are correlated with over 40–50% absolute lifetime risk of BC, and moderate-penetrance variants with over 20–25% absolute lifetime risk [9]. National organizations, including the National Comprehensive Cancer Network (NCCN), ASCO, and the American Society of Breast Surgeons (ASBrS), have published guidelines on the genetic assessment of hereditary BC among women with a BC diagnosis [1,29,30]. Although NCCN and ASCO determine eligibility for genetic testing based upon the family history of cancer, ancestry, age at diagnosis, and BC pathologic subtype, in 2019, ASBrS recommended that all women with a personal history of BC, regardless of family history of cancer and age at diagnosis, should be offered genetic testing with the aim of advising patients and their relatives on the risk of developing cancer in the future [9]. Following the 2013 US Supreme Court ruling against gene patenting, genetic testing has become more accessible to patients. Consequently, the large availability of multigene panel testing [31] has produced a higher rate of detection of variants of uncertain significance (VUS) and of P or LP variants of low-moderate penetrance BC susceptibility genes [32,33]. This trend has caused uncertainty in the management of patients with the possible overestimation of BC risk and the inappropriate application of risk-reducing strategies [9]. While *BRCA1* and *BRCA2* are the most frequently mutated genes in families with a high risk of breast and ovarian cancer, panel testing can identify less common syndromes that can also confer hereditary cancer risks [34,35,36,37,38,39,40], such as Li-Fraumeni syndrome (*TP53* pathogenic variant), Cowden syndrome (*PTEN* pathogenic variant), Hereditary diffuse gastric cancer syndrome (*CDH1* pathogenic variant), and Peutz-Jegher syndrome (*STK11* pathogenic variant) [30]. The identification of pathogenetic variants of these genes can influence the management of BC patients in terms of high-risk screening and risk-reduction surgery as well as with regard to the therapeutic options related to surgery, radiation, and systemic therapies [41,42,43]. Indeed, identifying a *BRCA1* PV in a BC patient provides information on her elevated risk of both contralateral BC and ovarian cancer, with all the implications for how to manage those risks. Studies are underway to determine whether these patients might benefit from the inclusion of PARP inhibitors in their adjuvant therapy [28]. Likewise, the added value of PV genes’ identification in the clinical management of BC patients is exemplified by the relative contraindication of radiation in patients with TP53 pathogenic variants (associated with Li-Fraumeni Syndrome), due to their higher risk of developing radiation-induced secondary cancers [30]. The information obtained from genetic testing should be evaluated together with all the clinicopathological features specific to each case, such as age, family history, medical history, cancer biological traits, and the existing management guidelines [30]. Risk management guidelines depend on the specific gene bearing the mutations and, on its penetrance, with the corresponding applicable medical strategies varying accordingly. The fact that a hereditary pathogenic mutation that predisposes to BC has been identified does not necessarily mean that risk-reducing mastectomy is always indicated. The possible presence of a significant family history of BC should also be taken into consideration [29,30]. Since the landscape of inherited mutations in high- and moderate-penetrance genes is associated with a different relative risk of breast cancer, guidance and decision-making in screening and prevention strategies, such as chemoprevention and prophylactic surgery, vary based on the different genetic variants. 

## 3. Risk-Reducing Surgery

### 3.1. Risk-Reducing Surgical Strategy in High Risk Women: Conservative Mastectomy

In women with an increased risk of hereditary BC, cancer risk, morbidity, and mortality can be reduced using a variety of possible preventive options that include, in addition to healthy lifestyle choices, intensified imaging screening to detect tumors at an earlier stage, prophylactic surgeries, i.e., risk-reducing mastectomy and risk-reducing salpingo-oophorectomy, and chemoprevention [28].

After the discovery of the *BRCA1* and *BRCA2* genes in the 1990s, the first guidelines for the care of high-risk individuals with hereditary breast and ovarian cancer stated that “there was insufficient evidence to recommend for or against prophylactic surgery” [44,45]. 

From 1999 to 2004, four studies were published that compared BC outcomes between women who underwent prophylactic mastectomies and women at similar risk who did not undergo surgery [44,46,47,48,49,50]. These demonstrated a 90% or greater reduction in the risk of subsequent BC among women who underwent prophylactic surgery with no difference in overall survival. Several further observational studies confirmed these findings, and currently, NCCN states that “risk-reducing mastectomy provides a high degree of protection against breast cancer in women with *BRCA 1* and *2* mutations” [51], specifying that patients with Li–Fraumeni or Cowden syndrome should discuss risk-reducing strategies in a personalized approach. The 2021 St. Gallen Consensus Conference asserted that “both age and the individual preferences of women, reflecting their perceptions of risk and general comfort with the various approaches, are the key drivers of these choices” [52]. The panelists recommended prophylactic mastectomy based on the degree of penetrance of the gene and the age of the woman with a genetic diagnosis and also favored considering risk-reducing mastectomy for women harboring highly penetrant genes (e.g., *BRCA1*, *BRCA2*, *TP53*, and *PALB2*) and surveillance with mammography and magnetic resonance imaging (MRI) for women with intermediate-penetrance genes (e.g., *BARD1*, *CHEK2*, *CDH1*, *STK11*). For women with less penetrant gene mutations (such as *ATM*, *BRIP1*, *NF1*, *RAD51C*, *RAD51D*), the Panel strongly recommended surveillance without prophylactic mastectomy [52].

Scientific data suggest that 48–90% of women chose prophylactic surgery versus constant surveillance strategies with MRI [28,53]. Over time, risk-reducing mastectomy in genetic variant carriers has become increasingly conservative. As simple mastectomy evolved first into skin-sparing mastectomy (SSM) and then into nipple-sparing mastectomy (NSM), scientific studies also aimed at validating the oncological safety, in terms of survival and local recurrence, of the so-called “conservative mastectomy” [54] in a prophylactic setting, stressing the importance of respecting women’s body image and safeguarding their well-being while highlighting the role of immediate breast reconstruction.

The safety of NSM was investigated by several single-center studies that involved limited numbers of germline carriers [55,56,57] and received strong support from the largest multi-institutional study of NSM series ever conducted in *BRCA1/2* mutation carriers and authored by Jakub and Colleagues in 2018 [58]. They reported that, in 346 patients who underwent 548 risk-reducing NSMs, no ipsilateral BC occurred after prophylactic NSM with a median follow-up of 34 months. Despite the short follow-up period, the cumulative evidence revealed NSM as an appropriate risk-reducing procedure for patients with genetic variants [58]. Over the last two decades, NSM has emerged as the preferred choice over SSM thanks to improved cosmetic outcomes and increased patient satisfaction when NSM is carried out with immediate reconstruction in both oncological and risk-reducing settings [59]. Indeed, Muller et al. recently underlined the extremely low risk of cancer development following breast risk-reducing surgery as follows: specifically, out of 3716 cases of prophylactic NSMs, only 9 cases (0.2%) of BC exterior to the nipple-areola complex (NAC) and 1 case (0.004%) of BC within the NAC were reported [60]. To date, NSM appears to be a safe risk-reduction procedure for *BRCA* mutation carriers from an oncological point of view, as highlighted in a recent literature review, which reported low rates of new BCs, low rates of postoperative complications, and high levels of satisfaction and postoperative quality of life [61].

In *CDH1*, as well as in *PALB2* and in *TP53* mutation carriers, risk-reduction recommendations now suggest considering prophylactic mastectomies [62]. Recent studies have reported an association between *CDH1* germline mutations and lobular breast cancer. In the context of the so-called hereditary lobular BC, cancer risk management requires prophylactic mastectomy in the case of an important family history of BC aggregation [63]. 

Surgical risk reduction in patients with moderate-penetrance genes is generally not recommended given the low risk associated with these mutations and the limited amount of available data on outcomes [15,25].

### 3.2. Not Only Mastectomy: An Opportunity for Breast-Conserving Surgery in High Risk BC Patients

Prophylactic bilateral mastectomy effectively reduces BC risk in *BRCA* mutation carriers and is considered a suitable option for primary prevention by healthy carriers as well as carrier patients who have already developed BC. However, unlike the guidelines on risk management, those on the role of local or systemic treatment in women with hereditary BC are scarce [15].

Risk-reducing surgery is currently witnessing a growing impulse towards the inclusion of breast-conserving therapy (BCT), defined as breast-conserving surgery (BCS) combined with radiotherapy (RT), in the personalized and multidisciplinary approach to managing BC high-risk patients [64]. Indeed, the debate on what the optimal local therapy for women with *BRCA*-associated BC should be is still ongoing.

Mastectomy is a disfiguring procedure that may significantly damage the quality of life. Women fear its effects on body image and sexuality, as well as the loss of sensation in the reconstructed breasts [65,66,67]. Breast cancer and its treatments may inflict harmful consequences on overall women’s quality of life as follows [68]: a mastectomy creates an undoubted change in body image, with loss of the surrounding sensory nerves to the breast [69]. Indeed, an undesirable outcome of mastectomy is loss of sensation to the remaining skin flap, which may negatively impact on psycosexual and relational well-being [70].

Recent data reported improved self-esteem, satisfaction, and psychosocial well-being in women who underwent BCT compared to mastectomy [71,72,73], otherwise showing that the preservation of NAC in NSM, compared to its deferred reconstruction after SSM, improved satisfaction in the physical appearance [74], even if nipple sensation is largely or completely lost in most cases [75,76]. 

The BREAST-Q Sensation Module has been recently developed and validated to also measure breast sensation in the overall evaluation of outcomes of breast cancer treatments [69]. To date, the restoration of breast sensation is an evolving frontier in breast reconstruction [69] and attests to the growing consideration of the quality of life following mastectomy.

Although BCT is the option of choice for surgical treatment of early-stage sporadic BC, its oncological safety in *BRCA1/2* mutation carriers is currently an object of several extensive studies. Indeed, the choice of treatment for sporadic BC is influenced by factors that include tumor location, breast size, and patient preference [77]. In reference to sporadic BC, multiple prospective randomized trials have found equivalent patient survival rates after BCS and after mastectomy [64]. In particular, Veronesi et al. [78] and other authors [79,80,81] found that the 20-year overall survival rate did not differ in a statistically significant way (*p* = 0.1) between patients who had undergone BCS (58.3%) and those who had undergone mastectomy (58.8%).

Despite seeing a higher local recurrence rate, several large studies validated BCT since no differences in overall survival were found in comparison to mastectomy [82,83,84].

As no randomized controlled trial has yet directly compared BCS and mastectomy in *BRCA* mutation carriers, the American Society of Clinical Oncology (ASCO), the American Society for Radiation Oncology (ASTRO), and the Society of Surgical Oncology (SSO) have provided guidelines on the management of BC in patients with germline mutations in the *BRCA 1/2*, *PALB 2*, *CHEK 2,* or *ATM* gene [15], underlining that BCS followed by RT should not be excluded from multidisciplinary discussion, but should be offered to patients with newly diagnosed hereditary BC, due also to the possibility of including RT, except in the case of the TP53 mutation.

However, for *BRCA* mutation carriers with BC, the choice between BCT and mastectomy is an object of intense research because the oncologic outcomes of BCT are controversial. The debate also concerns the possible predictive value of radiotherapy in *BRCA* carriers, given the genome instability and consequent secondary carcinogenesis due to the homologous recombination deficiency caused by *BRCA* mutations [17], even if the clinical data are conflicting and inconclusive [85].

Scientific studies are currently comparing survival, risk of ipsilateral BC recurrence, and new primary BCs between *BRCA* mutation carriers that received BCT and those that underwent a mastectomy, with special attention to patients who also had prophylactic bilateral salpingo-oophorectomy and adjuvant systemic therapy [64].

## 4. A Breast-Conserving Approach to Hereditary Breast Cancer: The Impact on Outcomes

As recommended by ASCO/ASTRO/SSO guidelines [15,86], the best surgical strategy for high-risk BC patients should be selected based on several factors, which include the patient’s genetic risk, family history, age, co-morbidities, previous personal oncological history, BC biology, life expectancy, ability to undergo appropriate breast surveillance, as well as own preferences.

The Prospective Outcomes in Sporadic versus Hereditary breast cancer (POSH) study (Table 1) confirmed that in young BC patients’ overall survival did not differ between *BRCA* mutation carriers and non-carriers [16], suggesting that the oncological behavior of inherited BC could be similar to that of sporadic BC, also in terms of local recurrences, meaning both as real recurrence and as second primary BC.

Data on the best local therapy remains conflicting, especially those regarding the oncological success of BCT in high-risk gene carriers (Table 1 and Table 2).

In 2006, Pierce et al. compared outcomes in *BRCA 1/2* carriers BC patients treated with BCT with those in matched sporadic controls and demonstrated similar 10-year rates of ipsilateral breast tumor recurrence (IBTR), suggesting that the use of tamoxifen and bilateral oophorectomy were correlated with minor IBTR/new primary cancer risk and fewer contralateral BCs in mutation carriers [87].

In 2009, a single retrospective study reported an increased risk of IBTR after BCT in *BRCA1/2* mutation carriers in comparison to patients who had sporadic BC. The 10-year cumulative incidence of IBTR was found to be 27% in mutation carriers and 4% in sporadic controls (hazard ratio 3.9; 95% confidence interval 1.1–13.8; *p* = 0.03). In *BRCA1/2* mutation carriers, the risk of contralateral BC (CBC) was also found to be higher, with a 10-year cumulative incidence of 25% as opposed to the 1% found in sporadic controls (*p* = 0.03) [88]. Over time, several retrospective studies comparing BCT to mastectomy in high-risk BC patients showed an increased risk of local recurrence [89,90,91,92]. A meta-analysis of ten studies also confirmed a significantly higher risk of IBTR in *BRCA1/2* mutation carriers compared to non-carriers following BCS at a median follow-up greater than 7 years, but no difference was found for shorter follow-up periods [93].

Interestingly, in 2019, a cohort of 6484 women who had been diagnosed with invasive BC at <50 years and treated between 1970 and 2003 in 10 Dutch centers, was analyzed with the objective of investigating the effects of different types of surgery on BC prognosis in germline *BRCA1/BRCA2* mutation carriers compared with non-carriers [90]. The obtained results supported literature data reporting that BCT is a safe local treatment option to offer to *BRCA1* mutation carriers with invasive BC as follows [90]: after adjustment for potential confounders, overall survival rates in patients who performed BCT were similar to those in patients who received a mastectomy, both for BRCA1 mutation carriers (hazard ratio HR = 0.80, confidence interval CI = 0.42–1.51, *p* = 0.50) and for non-carriers ([HR] = 0.95, [CI] = 0.85–1.07, *p* = 0.41). For BRCA2, the number of cases was instead insufficient to draw conclusions. The rate of local recurrences after BCT did not differ between *BRCA1* carriers (10-year risk = 7.3%) and non-carriers (10-year risk = 7.9%) [90].

Moreover, a multi-institutional retrospective analysis by Bernstein-Molho and colleagues reported higher rates of new primary BC in *BRCA*-associated BC patients treated with BCT than in those treated with mastectomy, although there were no differences in regional or distant recurrences or in overall survival, suggesting BCT is an acceptable alternative to mastectomy in the treatment of *BRCA1/2*-associated BC, despite the higher lifetime risk of developing BC [92].

In the absence of clear overall survival advantage of mastectomy over BCT in *BRCA1*/2 mutation carriers, the decision to opt for BCT ought to be agreed upon with the patient and follow a multidisciplinary consultation that also considers the risk of ipsilateral breast tumor recurrence. Preoperative counseling plays a crucial role, as confirmed by a pooled analysis of over 3800 *BRCA* mutation carriers treated with either BCT or mastectomy that demonstrated superimposable outcomes in terms of disease-free, disease-specific, and overall survival with 96-month follow-up and the median age at BC diagnosis of 41 years [94]. At intermediate and long follow-up, a 4.5-times increased risk of loco-regional recurrence in the BCT group was instead described [94]. These findings agree with a recent review by Co et al. [77] who calculated overall survival at 5, 10, and 15 years from 18 different studies and found comparable values in BCS (88.7%, 89.0%, and 83.6% respectively) and mastectomy (83%, 86.0%, and 83.2% respectively). These authors also showed that BCS was associated with a greater rate of ipsilateral BC recurrence in *BRCA* mutation carriers, revealing that the pooled ipsilateral BC recurrence rates at 5, 10, and 15 years were higher in the BCS group (8.2%, 15.5%, and 23%, respectively) than in the mastectomy group (3.4%, 4.9%, and 6.4%, respectively) [77]. However, this analysis did not take into consideration recent studies on the same subject [90,92,95], one of which, authored by Huang et al., concluded to the contrary that BCT and mastectomy have similar local recurrence rates [95]. 

To date, a very recent meta-analysis [17] including four studies (three retrospective and one prospective cohort studies) with five cohorts and a total of 1254 *BRCA1*/2 mutated BC patients, concluded that BCT is associated with a significantly higher risk of local recurrence than mastectomy (HR 3.838, 95% CI = 2.376–6.201, *p* < 0.001). The pooled results revealed no significant impact of BCT on disease-free survival, metastasis-free survival, BC-specific survival, or overall survival. Patients who received mastectomy mostly underwent prophylactic contralateral mastectomy (BCT 16.5% vs. M 35.8%, *p* < 0.001), and most of those treated with BCT presented an early-stage BC with negative estrogen receptors. Regarding the similar rates of long-term disease-free survival, metastasis-free survival, BC-specific survival, and overall survival observed for BCT and mastectomy, which would appear to compensate for the significant difference in local recurrence rates, the authors underlined the potential protective effect of chemotherapy caused by the enhanced chemosensitivity of *BRCA* mutation carriers due to their homologous recombination deficiency [17].

The present narrative review confirms the scientific liveliness regarding the surgical approach in the hereditary BCs treatment panorama. Although characterized by the limitation of analyzing the most significant literature of the last two decades, beyond the intrinsic limits of each study included, it could offer a tool to guide clinicians in the multidisciplinary choice of the surgical option for such patients. 

The current era of personalized medicine requires more and more appropriate counseling on different surgical strategies to offer the best care. 

The increasingly sophisticated and accurate definition of evidence-based scientific recommendations in the clinical management of hereditary breast cancer might provide in the future a progressively specialized approach, also to rare genetic clinical entities, such as hereditary lobular breast cancer, a novel inherited cancer predisposition linked to germline *CDH1* mutation, currently object of extensive and significant research [96].

## 5. Conclusions

Surgical risk reduction, both for breast and ovarian cancer, remains a valid tool in managing cancer risk in women with increased genetic susceptibility [28]. However, the current debate over absolute risks of cancer development, contralateral BC risk, ideal timing of risk-reducing surgery, and optimal surgical oncological strategy for BC patients carrying *BRCA* and other high-penetrance genetic mutations is lively and wide-reaching. The multidisciplinary discussion about surgical risk reduction and the type of BC surgical treatment to perform should be personalized based on BC clinical and pathological features and family history, while also taking into account current international guidelines, recent scientific insights, and the patient’s personal preferences.

## Figures and Tables

**Table 1:** Cohort Studies reported.

Author, Year	Study Design	Endopoints	Outcome Data
Pierce et al., 2006 [87]	Retrospective cohort study	To analyse outcome of BCS and RT in *BRCA1/2* mutation carriers with BC versus that of matched sporadic controls	- 160 *BRCA1*/2 mutation carriers with BC matched with 445 controls with sporadic BC; - Median follow-up of 7.9 years; - No significant difference in IBTR overall between carriers and controls; 10- and 15-year estimates were 12% and 24% for carriers and 9% and 17% for controls, respectively ([HR], 1.37; *p* = 0.19); - Multivariate analyses for IBTR found *BRCA1/2* mutation status to be an independent predictor of IBTR when carriers who had undergone oophorectomy were removed from analysis (HR, 1.99; *p* = 0.04); - CBCs were significantly greater in carriers versus controls, (HR, 10.43; *p* = 0.0001); - Tamoxifen use significantly reduced risk of CBCs in mutation carriers (HR, 0.31; *p* = 0.05).
Garcia Etienne et al., 2009 [88]	Retrospective cohort study	To investigate cumulative incidence of IBTR and CBC in *BRCA1/2-*associated BC patients matched with sporadic BC	- In total, 54 women with *BRCA1/2*-associated BC treated with BCS and whole RT matched with 162 patients with sporadic BC; - Median follow-up was 4 years for both groups; - Ten-year cumulative incidence of IBTR of 27% for mutation carriers and 4% for sporadic controls (hazard ratio 3.9; 95% confidence interval 1.1–13.8; *p* = 0.03); - Ten-year cumulative incidence of CBC of 25% for mutation carriers and 1% for sporadic controls (*p* = 0.03).
Nilsson et al., 2014 [91]	Prospective cohort study	To compare LR and OS between *BRCA1/2* mutation carriers treated with BCT and carriers treated with M. Endpoints: LR, OS, BC death, and distant recurrence	- BCT associated with an increased risk of LR in univariable analysis (HR 4.0; 95 % CI 1.6–9.8) and in multivariable analysis; - No significant differences between BCT and M for OS, BC death, or distant recurrence; - *BRCA1*/2 mutation carriers treated with BCT have a high risk of LR, many of which are new primary breast cancers.
Copson et., 2018POSH Study [16]	Prospective cohort study	Primary outcome: OS for all *BRCA1* or *BRCA2* mutation carriers (*BRCA*-positive) versus all non-carriers (*BRCA*-negative) at 2 years, 5 years, and 10 years after diagnosis Prespecified subgroup analysis of OS in patients with triple-negative breast cancer	- Median follow-up of 8.2 years; - In total, 2733 women aged 40 years or younger at first diagnosis recruited; - No significant difference in overall survival between *BRCA*-positive and *BRCA*-negative patients in multivariable analyses at any of the following timepoints: At 2 years: 97.0% [95% CI 94.5–98.4] vs. 96.6% [95.8–97.3]; At 5 years: 83.8% [79.3–87.5] vs. 85.0% [83.5–86.4]; At 10 years: 73.4% [67.4–78.5] vs. 70.1% [67.7–72.3]; hazard ratio [HR] 0.96 [95% CI 0.76–1.22]; *p* = 0.76). - In total, 558 patients with triple-negative BC: - *BRCA* mutation carriers had better OS than non-carriers at 2 years (95% [95% CI 89–97] vs. 91% [88–94]; HR 0.59 [95% CI 0.35–0.99]; *p* = 0.047) but not 5 years (81% [73–87] vs. 74% [70–78]; HR 1.13 [0.70–1.84]; *p* = 0.62) or 10 years (72% [62–80] vs. 69% [63–74]; HR 2.12 [0.82–5.49]; *p*= 0.12).
van den Broek et al., 2019 [90]	Prospective cohort study	To investigate effects of the BCT and M on OS and BCSS and to address the risk of LR and ipsilateral second primary breast cancer in germline *BRCA1/BRCA2* mutation carriers compared with noncarriers.	- <50 years 5820 noncarriers, 191 *BRCA1* and 70 *BRCA2* mutation carriers BC patients; - Patients who received BCT had a similar OS compared with patients who received M, both in noncarriers (hazard ratio [HR] 1⁄4 0.95, confidence interval [CI] 1⁄4 0.85–1.07, *p* = 0.41) and *BRCA1* mutation carriers (HR 1⁄4 0.80, CI 1⁄4 0.42–1.51, *p* = 0.50); - Numbers for *BRCA2* were insufficient to draw conclusions; - The rate of LR BCT did not differ between *BRCA1* carriers (10-year risk = 7.3%) and noncarriers (10-year = 7.9%).
Huang et al., 2020 [95]	Retrospective cohort study	To compare the prognostic impact of BCT and MT both in *BRCA1/2* mutation carriers and noncarriers with BC	- 176 *BRCA1/2* mutation carriers and 293 noncarriers in Chinese population; - Patients who received BCT had a similar BC DFS compared with patients who received MT, both in *BRCA1/2* mutation carriers and noncarriers [HR *BRCA* = 1.17, confidence interval (CI):0.57–2.39, *p* = 0.68; HR noncarriers =0.91, CI: 0.47–1.77, *p* = 0.79, respectively); - Recurrence free survival after BCT did not differ from MT in *BRCA1/2* mutation carriers [BCT, 5-year cumulative recurrence-free survival (RFS) = 0.95, CI: 0.89–1.00; MT, 5-year cumulative RFS = 0.93, CI: 0.85–1.00], even better for BCT in noncarriers (BCT, 5-year cumulative RFS = 0.67, CI: 0.42–0.89; mastectomy, 5-year cumulative RFS = 0.83, CI: 0.71–0.95).
Bernstein-Molho et al., 2021 [92]	Retrospective cohort study	To investigate treatment outcomes in *BRCA1/2* mutation carriers with BC who were treated with MT alone, MT and PMRT, or BCT	- 255 BC patients with *BRCA1/2* germline mutations; - Median follow-up of 57.7 months; - No significant difference in overall survival was observed at the time of follow-up; - The IBTR cumulative rate was 9 of 76 (11.8%) in the non-PMRT cohort compared with 0 of 52 in the PMRT group (*p* = 0.01) and 6 of 127 (4.7%) in the BCT group (*p* = 0.06); - The cumulative incidences of IBTR at 5 and 10 years were 9.8% and 27.4%, respectively, in the non-PMRT group versus 2% and 11.3%, respectively, in the BCT group (*p* = 0.0183).

Abbreviations: BCS, breast-conserving surgery; RT, radiotherapy; BCT, breast-conserving therapy; BC, breast cancer; DFS, disease-free survival; HR, hazard ratio; KM, Kaplan–Meier; OS, overall survival; IBTR, ipsilateral breast tumor recurrence; CBC, contralateral breast cancer; M, mastectomy; LR, local recurrence; BCSS, breast cancer-specific survival; PMRT, post-mastectomy radiotherapy; MFS, metastasis-free survival.

**Table 2:** Systematic review and meta-analysis reported.

Author, Year	Study Design	Endopoints	Outcome Data
Valachis et al., 2014 [93]	Systematic review and meta-analysis	Ten studies investigated: - The oncological safety of BCT in *BRCA*-mutation carriers versus non-carriers; - The risk for CBC compared with non-carriers; - Potential risk factors for IBTR or CBC; - To grade these factors based on the level of evidence.	- No significant difference in IBRT between carriers and controls (RR 1.45, 95% CI 0.98–2.14); - Significant higher risk for IBRT in *BRCA*-mutation carriers observed in studies with a median follow-up ≥7 years (RR 1.51, 95% CI 1.15–1.98). CBCs were significantly greater in carriers versus controls (RR 3.56, 95% CI 2.50–5.08); - Use of adjuvant chemotherapy and oophorectomy associated with a significantly lower risk for IBR in *BRCA*-mutation carriers.
Co et al., 2020 [77]	Systematic review and meta-analysis	To critically evaluate LR rates after BCT and MT in *BRCA* mutation carriers from reported studies	- 16 studies included; - analysis of OS at 5, 10, and 15 years were comparable between BCS and MT (88.7%, 89.0%, and 83.6% with BCS and 83%, 86.0%, and 83.2% with mastectomy, respectively); - IBTR rates at 5, 10, and 15 years were higher in the BCS group (8.2%, 15.5%, and 23%, respectively) than in the MT group (3.4%, 4.9%, and 6.4%, respectively).
Davey et al., 2021 [94]	Systematic review and meta-analysis	To evaluate the oncological safety of combined BCT versus MT in *BRCA* mutation carriers following BC diagnosis	- 23 studies of 3807 patients; - Median age at diagnosis was 41 years; - Median follow up of 96 months; - An increased risk of LR was observed in patients treated with BCS (HR: 4.54, 95% Confidence Interval: 2.77–7.42, *p* < 0.001; - The risks of CBC (HR: 1.51, 95% CI: 0.44–5.11, *p* = 0.510), disease recurrence (HR: 1.16, 95% CI: 0.78–1.72, *p* = 0.470), disease-specific recurrence (HR: 1.58, 95% CI: 0.79–3.15, *p* = 0.200) and death (HR: 1.10, 95% CI: 0.72–1.69, *p* = 0.660) were equivalent for combined BCT and mastectomy.
Wang et al., 2022 [17]	Systematic review and meta-analysis	To evaluate the impact of BCT on local control and survival for BC with *BRCA* mutations.	- 4 studies with 5 cohorts and totally 1254 patients included; - BCT had a significant higher risk for LR than M (HR 3.838, 95% CI = 2.376–6.201, *p* < 0.001); - No significant impact of BCT on DFS, MFS, BCSS and OS.

Abbreviations: BCS, breast-conserving surgery; RT, radiotherapy; BCT, breast-conserving therapy; BC, breast cancer; DFS, disease-free survival; HR, hazard ratio; KM, Kaplan–Meier; OS, overall survival; IBTR, ipsilateral breast tumor recurrence; CBC, contralateral breast cancer; M, mastectomy; LR, local recurrence; BCSS, breast cancer-specific survival; PMRT, post-mastectomy radiotherapy; MFS, metastasis-free survival.

## Data Availability

The data presented in this study are available on request from the corresponding author.
